# Fibro‐Adipogenic Progenitors From Skeletal Muscle Enhance Microvascular Angiogenesis in a 3D Model

**DOI:** 10.1002/adbi.70162

**Published:** 2026-10-15

**Authors:** Christian M. Mendiondo, Andrew R. King, Felipe Morales, David Leyton‐Cifuentes, Theodore J. Pabst, Eric M. Brey, Christopher R. Rathbone

**Affiliations:** ^1^ Department of Biomedical Engineering and Chemical Engineering UT San Antonio San Antonio Texas USA; ^2^ UT San Antonio Joint Graduate Program in Biomedical Engineering UT San Antonio San Antonio Texas USA; ^3^ Institute of Regenerative Medicine UT San Antonio San Antonio Texas USA

**Keywords:** angiogenesis, brown adipogenesis, fibro‐adipogenic progenitors, skeletal muscle, tissue‐engineering

## Abstract

Skeletal muscle tissue is highly vascularized, and angiogenesis plays a key role in both muscle homeostasis and regeneration. Despite their importance in regulating various processes within skeletal muscle, little is known about the role of muscle‐resident fibro‐adipogenic progenitors (FAPs) in promoting microvascular angiogenesis. In order to better understand the effects of FAPs on angiogenesis, FAPs were co‐cultured with adipose‐derived microvascular fragments (MVFs) in three‐dimensional fibrin hydrogels. We further tested if pre‐culturing FAPs in brown adipogenic induction media (BAM) influences their ability to promote MVF angiogenesis. MVFs co‐cultured with control FAPs or BAM FAPs for 5 days exhibit robust microvascular network formation with significantly increased vessel percentage area and average vessel length compared to MVFs alone. FAPs cultured in BAM prior to co‐culture with MVFs alter vascular network development compared to MVFs co‐cultured with control FAPs by significantly increasing average vessel length. Collectively, these results indicate that FAPs are key regulators of microvascular angiogenesis and that BAM FAPs have an improved capacity to promote increases in average vessel length. The current findings support the transplantation of FAPs and MVFs and modulation of FAP pro‐angiogenic properties as potential therapeutic approaches for enhancing skeletal muscle angiogenesis in the context of injury and disease.

## Graphical abstract

Fibro‐adipogenic progenitors (FAPs) are skeletal muscle stem cells that reside between fibers and near capillaries. However, relatively few studies have investigated interactions between FAPs and the microvasculature. We demonstrate that FAPs enhance angiogenesis of microvascular fragments (MVFs) by promoting increases in vessel percentage area and average length, and that FAPs pre‐stimulated in brown adipogenic media further increase average vessel length.

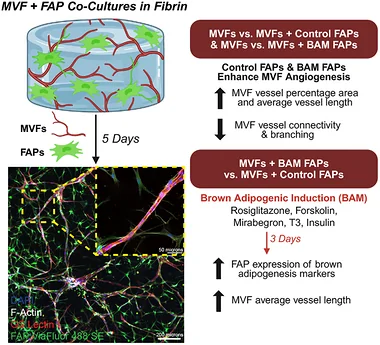

## 1 Introduction

Skeletal muscle is highly vascularized, and angiogenesis, the formation of new blood vessels from pre‐existing vasculature, plays a key role in both exercise adaptation and skeletal muscle regeneration. Crosstalk between the microvasculature and other cell types in the muscle microenvironment, such as satellite cells (SCs), immune cells, and fibroblasts, has been studied extensively over the years in order to better understand the cell‐to‐cell interactions underlying the process of angiogenesis in skeletal muscle [1, 2, 3, 4, 5, 6, 7, 8, 9]. For instance, SCs are in close association with capillaries, express abundant levels of vascular endothelial growth factor (VEGF), and promote angiogenic sprouting in vitro [1, 2, 3]. Reciprocally, capillaries have been shown to regulate SC self‐renewal and quiescence through a Dll4‐Notch pathway [4], and VEGF, a factor critical to the overall angiogenic response, significantly increases myoblast migration, proliferation, and resistance to apoptosis [5]. Reduced infiltration of monocytes/macrophages after cardiotoxin injury in mice lacking CC chemokine receptor 2 is associated with impaired skeletal muscle VEGF expression and capillary density [6]. Restorative M2 macrophages have also been shown to stimulate both angiogenesis and myogenesis through the secretion of oncostatin M [7], which is in line with previous findings identifying M2 macrophages as being pro‐angiogenic [8]. However, the influence of fibro‐adipogenic progenitors (FAPs) on the skeletal muscle microvasculature, especially regarding the process of angiogenesis, has received very little attention.

FAPs are muscle‐resident mesenchymal stem cells expressing platelet‐derived growth factor receptor α (PDGFRα), stem cell antigen‐1 (SCA‐1), and CD34 that reside in the interstitial space between muscle fibers and play a key role in skeletal muscle homeostasis and regeneration [10]. In muscle, FAPs are known for their influence on satellite cells and myogenesis, and their hallmark ability to adopt fibrogenic and white/brown adipogenic fates [10]. Interestingly, FAPs reside in close proximity to capillaries within skeletal muscle in vivo [11, 12], suggesting a role in the regulation of endothelial cells and vessel homeostasis. One previous study demonstrated that injection of undifferentiated FAPs after hindlimb ischemia injury promoted tissue revascularization and healing, as evidenced by reductions in mean vessel diameter, vessel permeability, and tissue fibrosis [13]. Importantly, it was also demonstrated that genetic ablation of PDGFRα^+^ FAPs prior to hindlimb ischemia impairs vessel organization and tissue revascularization, which correlated with an increase in fibrosis and overall tissue damage after injury. FAPs have also recently been shown to produce VEGFA, which influences muscle revascularization in an injury‐specific manner and is critical in the setting of ischemic damage [14].

Furthermore, FAPs differentiated toward a brown/beige adipocyte phenotype, characterized by the expression of uncoupling protein 1 (UCP‐1), are highly pro‐regenerative in a variety of skeletal muscle injuries, and the brown FAP phenotype may further impact angiogenesis [15, 16, 17]. For instance, one study investigating the pro‐regenerative effects of UCP‐1^+^ FAP transplantation following rotator cuff tears reported a marked increase in muscle capillary to fiber ratio [15]. Collectively, these findings suggest that FAPs modulate vessel formation in the context of injury and raise the question of whether the brown/beige phenotype enhances the pro‐angiogenic properties of FAPs. However, relatively few studies have investigated interactions between FAPs and microvessels and the effect of brown adipogenic induction. The overall lack of knowledge on the isolated role of FAPs in regulating angiogenesis and whether the brown adipogenic phenotype influences this process presents a knowledge gap in the field of skeletal muscle biology.

Accordingly, the objectives of the following study were to (1) determine the effects of FAPs on microvascular angiogenesis in a 3D cell culture model and (2) investigate whether inducing FAPs toward a brown adipogenic phenotype influences their pro‐angiogenic attributes. The model established and utilized in this study may serve as a powerful tool for future investigations that want to perform high‐throughput screening of how microvascular angiogenesis is influenced by FAPs derived from diseased or injured skeletal muscle, as well as those subjected to other forms of environmental or pharmacological stimuli.

## 2 Results

### 2.1 Experimental Design and FAP Characterization by Flow Cytometry

FAPs purified via magnetic‐activated cell sorting (MACS) were pre‐cultured in standard growth media (GM) or brown adipogenic induction media (BAM) for 3 days, then co‐cultured with freshly isolated adipose‐derived microvascular fragments (MVFs) in fibrin hydrogels for 5 days, at which point vascular networks were imaged via confocal microscopy and analyzed (Figure 1A,B). Flow cytometry confirmed the purity of the MACS‐sorted FAP population, with 98.5% and 97.1% of cells expressing PDGFR⍺ and SCA‐1, respectively (Figure 1C). Single cells and gating thresholds for fluorescence were determined with forward and side scattering and with negative controls, respectively.

**FIGURE 1:**
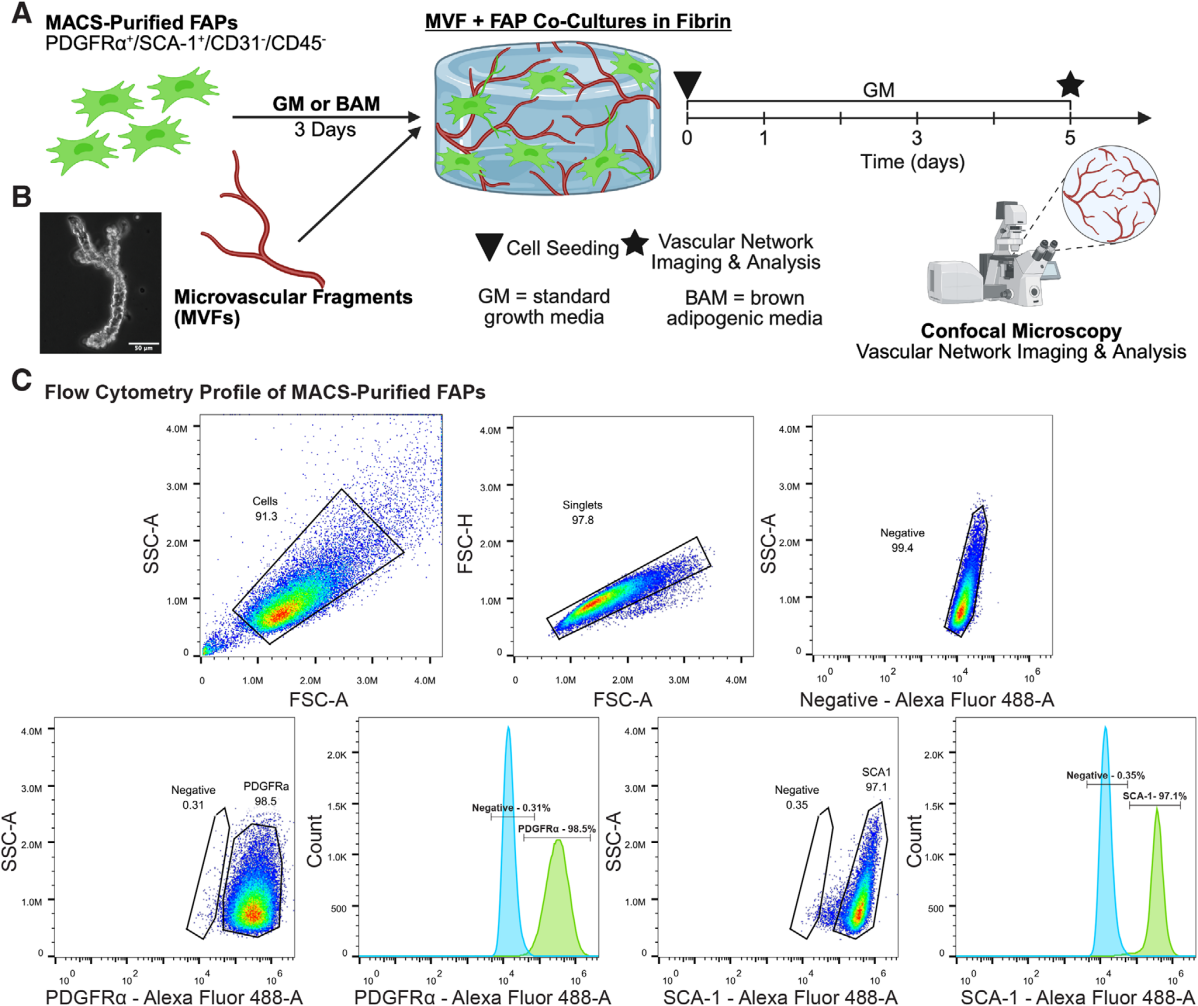
Experimental Design and Characterization of FAPs and Freshly Isolated MVFs. (A) Schematic representing the study design (Created with BioRender.com). (B) Representative phase contrast image of a single microvascular fragment immediately after isolation. (C) Representative flow cytometry gating strategy and scatterplots of PDGFRα and SCA‐1 expression in FAPs after magnetic‐activated cell sorting.

### 2.2 FAPs Co‐Cultured With MVFs Localize to Vessels and Enhance Angiogenesis

MVFs in fibrin hydrogels for 1 day expressed GS Lectin I and possessed a morphology characteristic of isolated microvessel segments (Figure 2A,D). When MVFs were cultured for 1 day with FAPs pre‐labeled with cell tracer ViaFluor 488 SE (VF488), FAPs acquired a perivascular position as evidenced by the presence of VF488^+^ FAPs (green) in direct contact with MVF structures (Figure 2C,F). After 5 days, MVFs co‐cultured with FAPs in fibrin hydrogels also exhibited significantly more robust vascular network development when compared to MVFs alone (Figure 2G,J), which assemble into short, dense, and highly interconnected networks. Specifically, MVFs in the presence of FAPs formed robust networks comprised of visibly longer and thicker vessels in which lumens were frequently observed (Figure 2I,L). In the co‐culture systems, VF488^+^ FAPs often localized near the vessel periphery (Figure 2I,L), indicating that at least some FAPs had acquired a perivascular position similar to that observed in vivo [11, 12, 13]. As expected, vessel networks did not form at day 1 or day 5 in fibrin gels containing only FAPs, which is clear given the absence of GS Lectin‐I^+^ vessel structures and the uniform distribution of VF488^+^ FAPs (Figure 2B,E,H,K).

**FIGURE 2:**
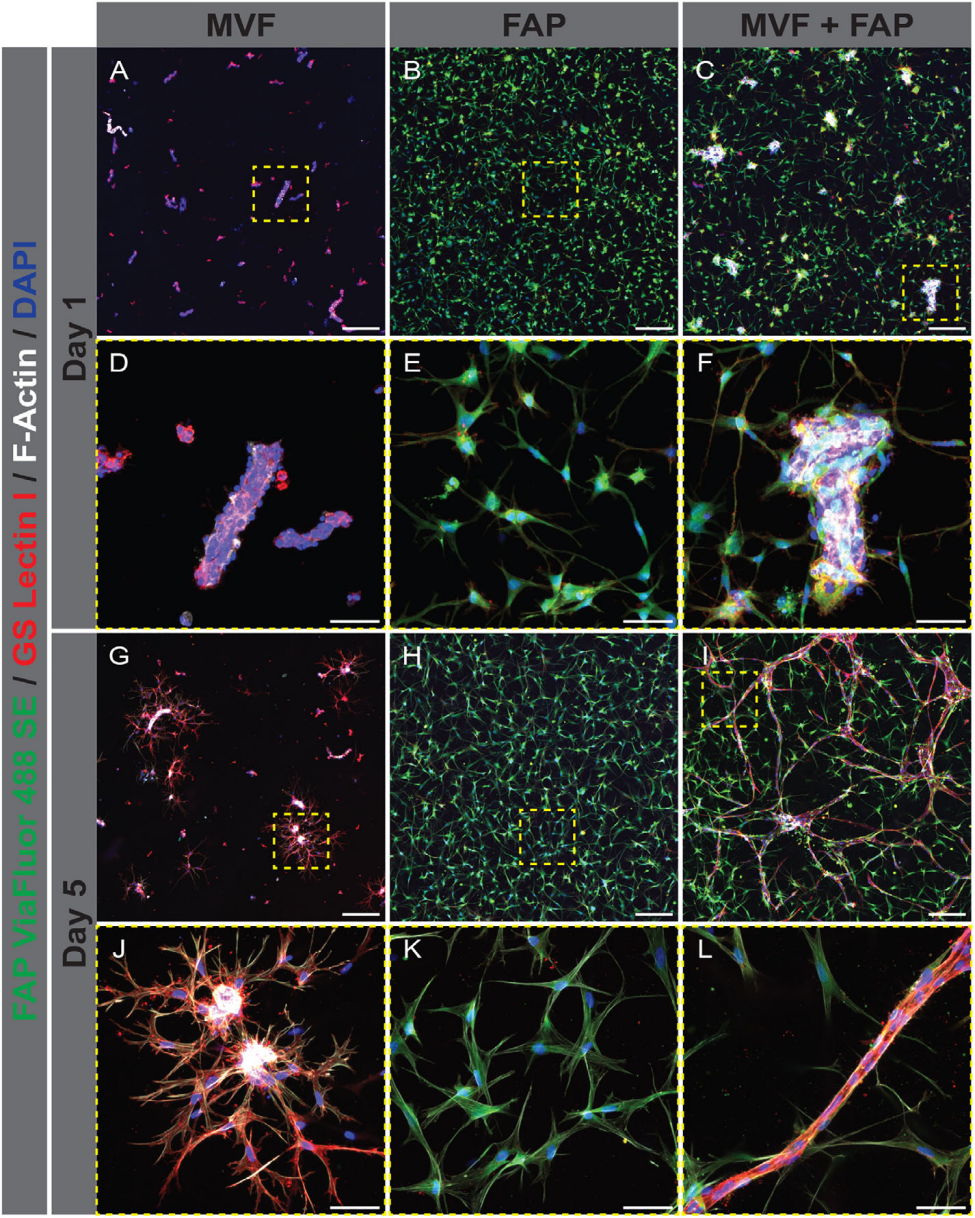
Immunofluorescence analysis of microvascular fragments co‐cultured with ViaFluor 488 SE pre‐labeled FAPs in fibrin hydrogels for 1 and 5 days. Representative confocal images of MVF, FAP, or MVF + FAP co‐cultures after (A–F) 1 day and (G–L) 5 days stained with GS Lectin I to visualize vascular network formation relative to FAP content and localization (GS Lectin I‐Red, FAP ViaFluor 488 SE‐Green, F‐Actin‐White, DAPI‐Blue, Scale Bar = 200 µm for A‐C and G‐I, Scale Bar = 50 µm for D‐F and J‐L).

### 2.3 FAPs Pre‐Stimulated With BAM Express Key Genetic Markers Associated With Brown Adipogenic Induction

Previous studies show that FAPs differentiated toward a brown/beige adipocyte phenotype are extremely pro‐regenerative, and one aspect of this may be linked to their ability to enhance angiogenesis within skeletal muscle [15, 16, 17]. To determine whether the ability of FAPs to promote angiogenesis could be influenced by activating their brown adipogenic programming, FAPs were pre‐stimulated with brown adipogenic induction media (BAM FAPs) or control growth media (CTRL FAPs) for 3 days prior to being co‐cultured with MVFs. Although both types of FAPs appeared morphologically similar under phase contrast microscopy (Figure 3A), real‐time quantitative polymerase chain reaction (RT‐PCR) analysis revealed that BAM FAPs expressed *peroxisome proliferator‐activated receptor gamma* (*PPARγ*) (BAM FAPs: 9.29 × 10^−8^ ± 2.02 × 10^−8^; Figure 3B), a key genetic regulator of FAP adipogenesis that was not reliably detected in CTRL FAPs [18]. Most importantly, BAM FAPs also possessed significantly higher expression (∼13.9 fold) of the canonical brown adipocyte thermogenesis gene *UCP‐1* relative to CTRL FAPs (*p* = 0.0022, Figure 3C). BAM FAPs and CTRL FAPs did not differ significantly in the expression of mitochondrial biogenesis gene *peroxisome proliferator‐activated receptor gamma coactivator 1‐alpha* (*PGC‐1α*) (∼2.2 fold, *p* = 0.0605, Figure 3D), despite a slightly upward trend in BAM FAPs. Taken together, these data confirm that BAM FAPs were in the process of being induced toward a brown/beige adipocyte phenotype.

**FIGURE 3:**
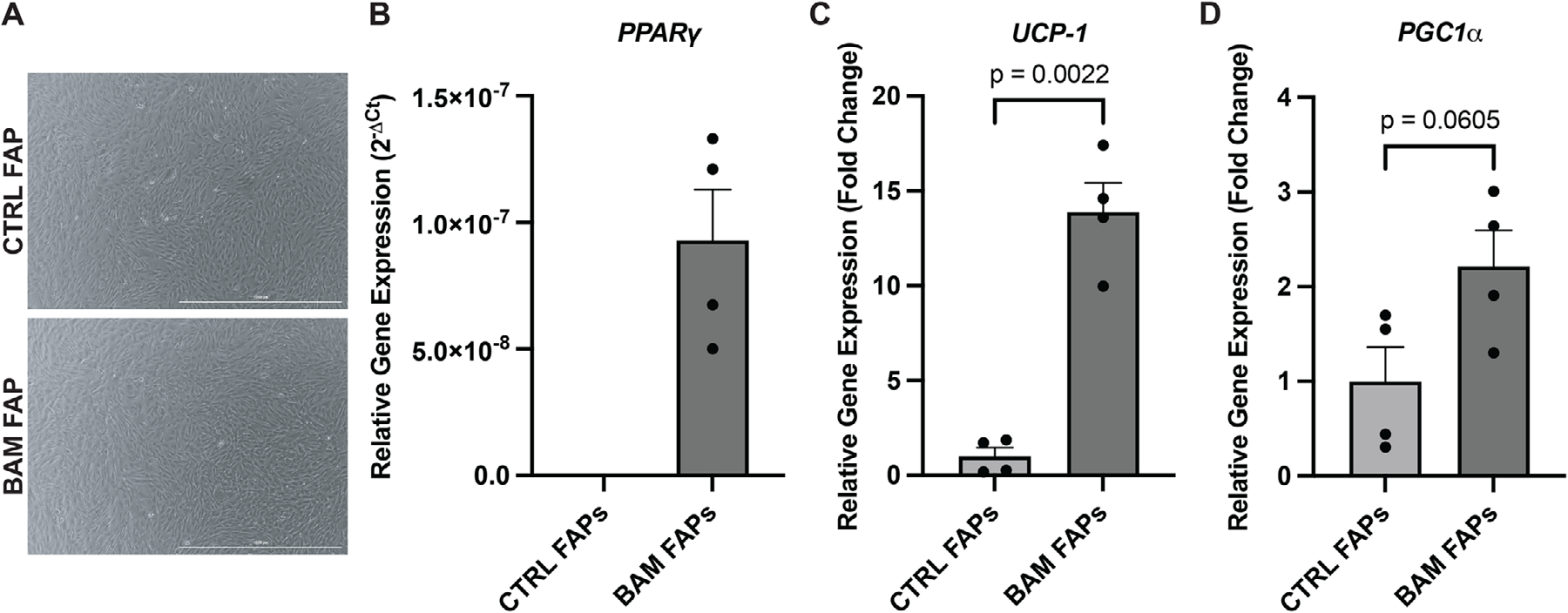
Phase contrast and RT‐PCR characterization of CTRL FAPs and BAM FAPs. (A) Representative phase contrast images of CTRL FAPs and BAM FAPs (Scale Bar = 1000 µm). (B–D) Relative expression of key brown adipogenesis genes, including *PPARγ*, *UCP‐1*, and *PGC‐1α*. Results are reported as mean ± standard error. *p* values < 0.05 indicate statistical significance.

### 2.4 CTRL FAPs and BAM FAPs Enhance Vascular Network Development of MVFs, With BAM FAPs Significantly Increasing Average Vessel Length

Quantitative analysis of vascular network images with AngioTool revealed that MVFs co‐cultured with CTRL FAPs and BAM FAPs had significant increases in vessel percentage area (MVF: 11.9 ± 1.37; MVF + CTRL FAPs: 19.5 ± 0.766, *p* = 0.0036; MVF + BAM FAPs: 23.7 ± 1.21, *p* = 0.0002, Figure 4B), and average vessel length (MVF: 0.727 ± 0.134 mm; MVF + CTRL FAP: 2.75 ± 0.102 mm, *p* = 0.0469; MVF + BAM FAP: 4.84 ± 0.732 mm, *p* = 0.0009, Figure 4D) compared to MVFs alone. MVFs grown in the presence of CTRL FAPs and BAM FAPs also had significant decreases in total number of junctions (MVF: 335 ± 41.90; MVF + CTRL FAP: 136 ± 11.20, *p* = 0.0006; MVF + BAM FAP: 184 ± 12.0, *p* = 0.0036, Figure 4E), junctions density (MVF: 176 ± 23.50; MVF + CTRL FAP: 61.1 ± 4.85, *p* = 0.0003; MVF + BAM FAP: 81.0 ± 4.73, *p* = 0.0012, Figure 4F), total number of endpoints (MVF: 245 ± 24.6; MVF + CTRL FAP: 118 ± 9.24, *p* = 0.0005; MVF + BAM FAP: 133 ± 8.08, *p* = 0.0012, Figure 4G), and mean lacunarity (MVF: 0.978 ± 0.162; MVF + CTRL FAP: 0.509 ± 0.0329, *p* = 0.0093; MVF + BAM FAP: 0.390 ± 0.0368, *p* = 0.0025, Figure 4H). Importantly, MVFs co‐cultured with BAM FAPs exhibited significant increases in average vessel length (MVF + BAM FAP: 4.84 ± 0.732 mm vs. MVF + CTRL FAP: 2.75 ± 0.102 mm, *p* = 0.0289; Figure 4D) compared to CTRL FAPs. While not statistically significant, vessel percentage area (MVF + BAM FAP: 23.7 ± 1.21 vs. MVF + CTRL FAP: 19.5 ± 0.766, *p* = 0.0559, Figure 4B) and total vessel length (MVF + BAM FAP: 22.7 ± 1.08 mm vs. MVF + CTRL FAP: 17.7 ± 0.984 mm, *p* = 0.0612, Figure 4C) trended upward in BAM FAPs compared to CTRL FAPs. Collectively, these data demonstrate that both CTRL FAPs and BAM FAPs significantly promote increases in MVF vessel percentage area and average vessel length, while decreasing connectivity and overall network branching. Furthermore, BAM FAPs promote the formation of vascular networks with significantly increased average vessel length.

**FIGURE 4:**
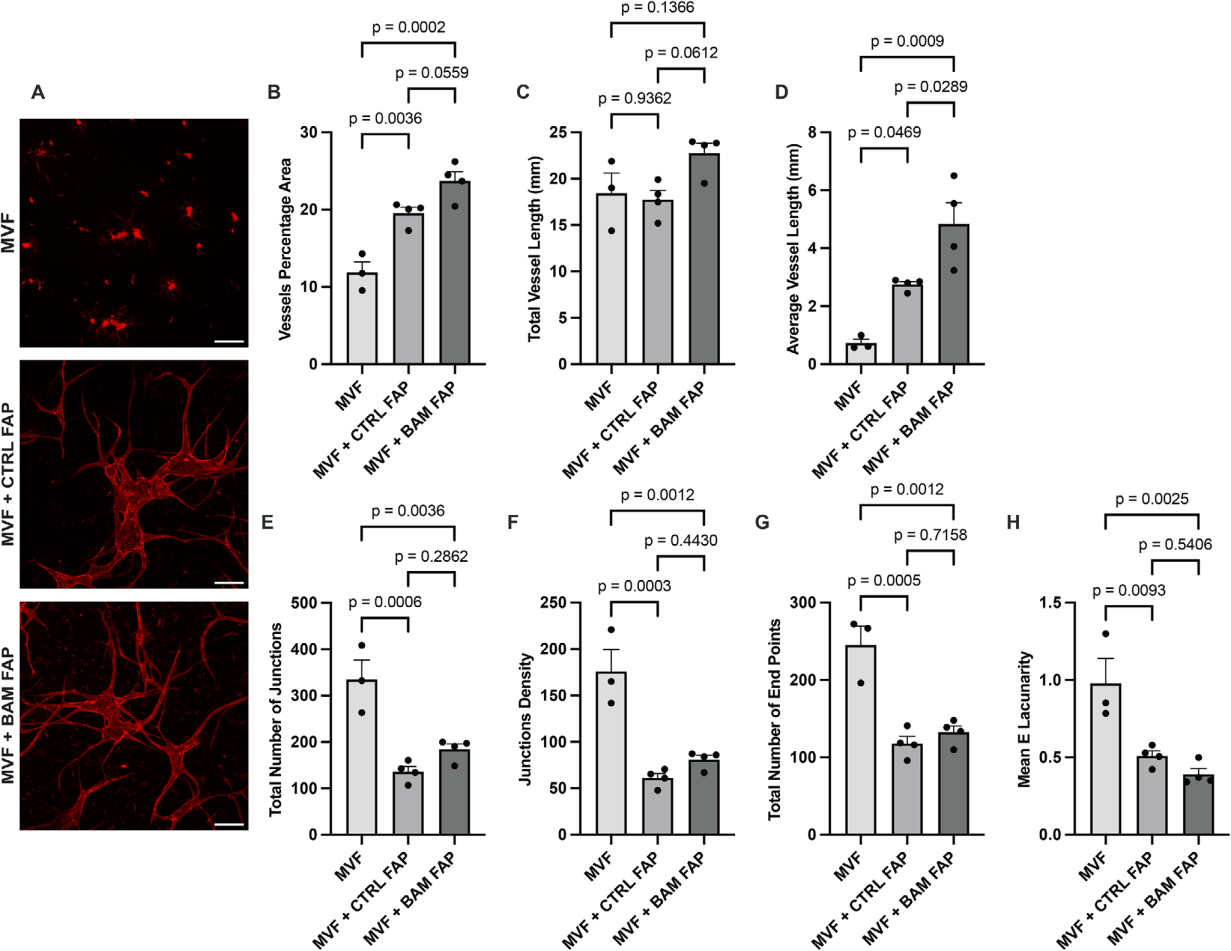
Immunofluorescence and quantitative analysis of vascular networks from MVFs and MVFs co‐cultured with CTRL FAPs and BAM FAPs after 5 days. (A) Representative confocal images of MVFs, MVFs + CTRL FAPs, and MVFs + BAM FAPs stained with GS Lectin I (red) to visualize vascular network formation (Scale Bar = 200 µm). (B) Vessel percentage area (C) Total vessel length (D) Average vessel length (E) Total number of junctions (F) Junction density (G) Total number of end points (H) Mean E Lacunarity. Results are reported as mean ± standard error. *p* values < 0.05 indicate statistical significance.

## 3 Discussion

FAPs are found in close proximity to capillaries in skeletal muscle and are critical for tissue revascularization after ischemic injury [11, 12, 13, 14]. Despite this knowledge, the influence of FAPs on microvessels at a steady state in the absence of injury is significantly understudied, especially in a physiologically relevant system that recapitulates the process of angiogenesis in vivo. Previous studies have not fully determined the extent to which changes in vascularization are a direct result of FAPs or rather an indirect effect of their influence on other aspects of regeneration within the muscle microenvironment. In the present study, we observe that FAPs localize to MVFs on day 1 and day 5 during their influence on vascular network formation, which is in line with their position in vivo and resembles previous findings that mesenchymal stem cells (MSCs) derived from skeletal muscle and other tissues acquire a perivascular position and are required for the generation of vascular networks when co‐transplanted with endothelial cells (ECs) in vivo [19]. These results indicate that FAPs establish direct cell contact with isolated microvessels and adopt a perivascular position as they facilitate the process of angiogenesis.

We further investigated whether control FAPs and those induced toward a brown/beige phenotype differ in their ability to enhance angiogenesis when co‐cultured with MVFs. Specifically, it was observed that both CTRL FAPs and BAM FAPs increase average vessel percentage area and average vessel length, while simultaneously decreasing overall connectivity and sprouting of the vascular network. These findings are similar to a previous study reporting that human umbilical vein endothelial cells (HUVECs) co‐cultured with either single‐passage FAPs cultured in 20% O_2_ or unpassaged FAPs cultured in 5% O_2_ increased mesh area and total branching when compared to HUVECs alone [13]. Importantly, we also show that BAM FAPs possess an enhanced ability to promote increases in MVF average vessel length compared to MVFs + CTRL FAPs and MVFs alone. Although not statistically significant, slightly upward trends in vessel percentage area and total vessel length were also observed in MVFs + BAM FAPs compared to MVFs + CTRL FAPs. These findings are in alignment with a previous study showing that transplantation of beige FAPs expressing UCP‐1 significantly increased supraspinatus capillary to fiber ratio when administered in tandem with a delayed repair 2 and 6 weeks after the initial rotator cuff tear [15]. Furthermore, these transplanted FAPs were found clustered around blood vessels, which is in accordance with both the observations from our FAP cell tracing experiments and previously published research on FAPs residing in close proximity to capillaries within muscle. It is important to note that MVFs are derived from adipose tissue and therefore the findings herein related to FAP‐mediated angiogenesis are not necessarily specific to skeletal muscle. Therefore, future studies should validate these findings by performing similar experiments with primary skeletal muscle endothelial cells or microvessels. Nevertheless, the results from our study demonstrate that FAPs serve as key regulators of angiogenesis and that brown adipogenic induction enhances their ability to facilitate increases in average vascular length. However, the exact mechanisms by which FAPs modulate angiogenesis warrant further investigation by future studies.

Although both CTRL and BAM FAPs significantly enhance MVF angiogenesis, we did observe that MVFs co‐cultured with BAM FAPs displayed an increase in average vessel length compared to MVFs + CTRL FAPs and MVFs alone. There are several possible explanations for the increase in average vessel length in the MVFs co‐cultured with FAPs pre‐stimulated with BAM. Although the 3‐day FAP stimulation performed in our study may not have been long enough to induce adipogenesis, brown/beige adipocytes are known to be highly vascularized compared to white adipose tissue and produce VEGF‐A [20, 21]. Interestingly, adeno‐associated virus‐mediated overexpression of VEGF in retroperitoneal white adipose tissue significantly increased brown markers UCP‐1 and PGC‐1α [22], while adipose‐specific ablation of VEGF significantly decreased UCP‐1 and Dio2 expression and impaired thermogenic capacity [23]. Collectively, these studies suggest a tightly coordinated relationship between the brown/beige phenotype and angiogenic programming, which may at least in part explain the increased average vessel length in MVFs co‐cultured with BAM FAPs (Figure 4A,D). Human FAPs isolated from patients with partial and full‐thickness rotator cuff tears and subjected to a brown adipogenic stimulus have also been shown to express increased levels of insulin‐like growth factor 1 (IGF‐1) and follistatin [24], both of which are implicated in promoting angiogenesis within skeletal muscle [25, 26, 27]. In fact, MVFs stimulated with IGF‐1 have an accelerated rate of vascularization and incorporation in vivo [28]. However, the effects of brown/beige stimulation on FAP expression of other paracrine factors and cell contact‐dependent processes related to angiogenesis have yet to be determined. While the exact mechanisms by which FAPs enhance angiogenesis should be further investigated, findings from the current study indicate that pre‐stimulation of FAPs with BAM promotes the formation of vascular networks with increased average vessel length compared to MVFs co‐cultured with CTRL FAPs and MVFs alone.

The significant influence of FAPs on MVF angiogenesis has exciting implications for cell‐based therapies and for the study of angiogenesis in skeletal muscle disease and injury. MVFs have been used extensively for regenerative medicine and cell therapy applications [29, 30, 31, 32]. In the context of skeletal muscle ischemic injury, MVF transplantation has recently been shown to significantly improve blood flow, increase capillary density, and alleviate tissue necrosis after hindlimb critical limb ischemia [33]. The finding that FAPs increase MVF angiogenesis indicates that MVFs and FAPs may serve as a more potent combinatorial cell therapy to induce angiogenesis after skeletal muscle ischemic insults such as ischemia‐reperfusion injury and critical limb ischemia. Future studies should also investigate how FAPs from diseased muscle differ in their ability to influence angiogenesis. This is a particularly interesting research avenue for skeletal muscle conditions where both FAP dysregulation and microvascular deficiencies are present. One example is muscular dystrophy, where skeletal muscle is characterized by the accumulation of pro‐fibrotic FAPs [34, 35], as well as a reduced capillary density and altered microvascular network organization [36]. It is plausible that FAPs play a role in the microvascular impairments observed in muscular dystrophy, which would align with previous findings that satellite cells obtained from dystrophic *mdx* mice have reduced gene expression of VEGF and hypoxia‐inducible factor 1‐alpha (HIF1α) and a diminished ability to stimulate MVF angiogenesis [37]. Another example is type 2 diabetes, where human skeletal muscle contains higher numbers of CD90^+^ FAPs that are more prone to rapid cell cycle entry, clonogenicity, and extracellular matrix (ECM) production [38]. Unlike their CD90^−^ counterparts, this subpopulation of FAPs is not enriched in gene sets related to angiogenesis and regulation of endothelial cell proliferation and migration [38], which is interesting given that reduced capillary density is a key feature of type 2 diabetic skeletal muscle [39]. However, the extent to which aberrant FAP behaviors drive impairments in skeletal muscle vascularization in these diseases and others warrants further investigation.

## 4 Conclusion

In this study, we demonstrate that FAPs are key regulators of microvascular angiogenesis and that FAPs induced toward a brown adipogenic phenotype facilitate the development of vascular networks with increased average vessel length. Collectively, these findings suggest that co‐transplantation of MVFs and FAPs and targeted enhancement of FAP‐mediated angiogenesis may be novel therapeutic approaches to improve vascularization in skeletal muscle injuries and diseases.

## 5 Experimental Section/Methods

### 5.1 Animals

Experiments were conducted using FAPs isolated from retired male breeder Lewis (Charles River) and MVFs isolated from retired male Sprague Dawley (Envigo) rats. All animals were housed in a temperature‐controlled environment with a 12‐h light‐dark cycle and fed ad libitum. This study was conducted in compliance with the Animal Welfare Act and the Implementing Animal Welfare Regulations, in accordance with the principles of the Guide for the Care and Use of Laboratory Animals. All animal procedures were approved (Protocol RA‐076) by the Institutional Animal Care and Use Committee at The University of Texas at San Antonio.

### 5.2 FAP Isolation and Magnetic‐Activated Cell Sorting

FAPs were isolated from Lewis rats (Charles River) using a modified version of the methods previously published by Te et al. [40]. Briefly, hindlimb skeletal muscle tissues (tibialis anterior, gastrocnemius, plantaris) were minced with sterile scissors, enzymatically digested in 6 mL Dulbecco's Modified Eagle medium (DMEM) + 1% penicillin‐streptomycin (P/S) containing 520 U/mL collagenase II (17101015, Gibco) for 1 h at 37°C in an orbital shaking water bath, and then with 580 U/mL collagenase II and 0.035 U/mL dispase II (17105041, Gibco) for 30 min at 37°C in an orbital shaking water bath. Samples were passed through an 18G needle equipped with a 10 mL syringe for 10 cycles, passed through a 40 µm cell strainer, then centrifuged (400 g × 15 min) to obtain a pellet of muscle‐derived cells. Cell pellets were incubated with 1 mL of RBC Lysis buffer (00‐4333‐57, Invitrogen) for 7 min, then completed to 10 mL with GM and centrifuged at 400 g × 15 min before seeding in four 150 mm Petri dishes (130183, Thermo Fisher Scientific). Following a 24‐h pre‐plate to separate non‐adherent satellite cells and their myogenic progeny, the adherent cells (P0) were expanded for a single passage, then subjected to MACS to obtain a population of PDGFRα^+^/SCA‐1^+^/CD31^−^/CD45^−^ FAPs. Briefly, the adherent cells were resuspended in 200 µL of DMEM + 2% FBS and incubated with antibodies (1 µL per 2.0 × 10^6^ cells) against PDGFRα (ab203491, Abcam), SCA‐1 (AB4336, Millipore Sigma), CD31‐PE (130‐116‐538, Miltenyi Biotec), and CD45‐PE (130‐126‐312, Miltenyi Biotec) on ice for 30 min. For CD31/CD45 depletion, cells were incubated with 10 µL of Anti‐PE MicroBeads (130‐048‐801, Miltenyi Biotec) on ice for 30 min, washed twice in 1 mL of MACS buffer + 0.5% bovine serum albumin (BSA) via centrifugation (2000 rpm × 3 min at 4°C), and subjected to negative selection with LD Columns (130‐042‐901, Miltenyi Biotec) according to manufacturer protocols. For enrichment of PDGFRα and SCA‐1 cells, the flow‐through from the previous step was incubated with 10 µL of Anti‐Rabbit IgG MicroBeads (130‐048‐602, Miltenyi Biotec) on ice for 30 min, washed twice in 1 mL MACS buffer + 0.5% BSA via centrifugation (2000 rpm x 3 min at 4°C), and subjected to positive selection with MS Columns (130‐042‐201, Miltenyi Biotec) according to manufacturer protocols. After MACS, FAPs were expanded in standard GM comprised of DMEM, 20% fetal bovine serum (FBS), 1% P/S, and 0.2% MycoZap. All experiments were conducted with MACS‐purified FAPs between passage 3 and passage 4.

### 5.3 Flow Cytometry

Expression of PDGFRα and SCA‐1 on cells that were subjected to the FAP MACS sorting strategy was verified using flow cytometry. Briefly, 1.0 × 10^6^ cells were collected via centrifugation (1500 g × 5 min) in several 1.5 mL microcentrifuge tubes and fixed with 250 µL of 4% paraformaldehyde for 5 min at room temperature. Cells were washed twice with 0.5 mL of phosphate‐buffered saline (PBS) by centrifugation (1500 g × 5 min), blocked with 500 µL of 10% goat serum in PBS for 30 min at 4°C, then incubated in blocking solution with antibodies against PDGFRα (1:50, ab203491, Abcam) and SCA‐1 (1:50, AB4336, Millipore Sigma). Cells were washed 3 times with PBS via centrifugation (8000 rpm × 2 min) and separately incubated in blocking solution with an Alexa Fluor 488‐conjugated goat anti‐Rabbit IgG secondary antibody (1:500, A11008, Thermo Fisher Scientific) for 30 min at 4°C. Cells were washed 3 times with PBS via centrifugation (8000 rpm × 2 min), resuspended in 200 µL of PBS, and stored protected from light at 4°C until analysis. Flow cytometry analysis was performed at the UTSA Cell Analysis Core with a Cytek Aurora spectral flow cytometer (Cytek Biosciences) using a minimum of 20,000 events per sample. Flow cytometry gates were sequentially established to exclude cell debris, identify singlets, and determine PDGFR⍺ and SCA‐1 expression independently relative to unstained negative controls.

### 5.4 MVF Isolation

MVFs were utilized in this study as a tool to evaluate the effects of FAPs on 3D vascular network formation. MVFs were isolated from epididymal, inguinal, and subcutaneous adipose tissue depots of retired male Sprague Dawley (Envigo) rats as described previously [41, 42, 43]. Briefly, adipose tissues were subjected to digestion with 3.86 mg/mL collagenase type I (NC9633623, Worthington Biochemical Corporation) for 15–20 min at 37°C in an orbital shaking water bath and then centrifuged (400 g × 5 min) to obtain a pellet containing adipose‐derived cells and MVFs. The pellet was resuspended in PBS containing 0.1% BSA (Sigma–Aldrich) and serially filtered through 500 and 37 µm filters to eliminate debris and reduce contamination with individual cells, respectively. MVFs were counted and centrifuged prior to resuspension in fibrinogen media as described below. All experiments were conducted with freshly isolated MVFs.

### 5.5 FAP Brown Adipogenic Induction

Prior to co‐culture with MVFs, FAPs were cultured in standard GM or brown adipogenic induction media (BAM; DMEM/F12, 20% FBS, 1% P/S, 0.2% MycoZap, 1 µM Rosiglitazone, 10 µM Forskolin, 1 µM Mirabegron, 20 nM triiodothyronine (T3), and 10 µg/mL Insulin) for 3 days to generate CTRL FAPs and BAM FAPs, respectively. GM was supplemented with the same final concentration of dimethyl sulfoxide (DMSO) as BAM to control for the DMSO used to solubilize Rosiglitazone, Forskolin, Mirabegron, and T3 in BAM. A similar formulation has previously been used to induce brown adipogenic differentiation [42].

### 5.6 RNA Isolation and Quantitative RT‐PCR

CTRL FAPs and BAM FAPs grown in 24‐well plates (3 wells/group) were lysed with 0.5 mL of TRIzol (Thermo Fisher Scientific), and RNA was isolated using a Qiagen RNeasy Mini Kit (Qiagen) according to manufacturer protocols. RNA concentration of each sample was measured on a Take3 microvolume plate (BioTek), at which point 150 ng of RNA was converted to cDNA using an iScript cDNA synthesis kit (Bio‐Rad). RT‐PCR was performed with a CFX96 Touch Real‐Time PCR Detection System (Bio‐Rad) using iTaq Universal SYBR Green Supermix (Bio‐Rad) and the predesigned primer sequences (Sigma–Aldrich) listed in Table 1. Relative gene expression was calculated for each group using the 2^−ΔCt^ method and expressed as fold change when possible, with 18S expression serving as the housekeeping gene.

**TABLE 1:** RT‐PCR Primer Sequences.

Gene	Forward Primer 5ʹ – 3ʹ	Reverse Primer 5ʹ – 3ʹ
*PPARγ*	ACCCAGAGCATGGTGCCTTCG	TGGTGGGCCAGAATGGCATCTCT
*UCP‐1*	GTGAAGGTCAGAATGCAAGC	AGGGCCCCCTTCATGAGGTC
*PGC1α*	GTGCAGCCAAGACTCTGTATGG	GTCCAGGTCATTCACATCAAGTTC

### 5.7 MVF‐FAP Co‐Culture

MVFs (20,000 MVFs/mL), FAPs (5 × 10^5^ cells/mL), or MVFs + FAPs (20,000 MVFs/mL; 5 × 10^5^ cells/mL) were resuspended in 20 mg/mL fibrinogen media solubilized in DMEM. MVFs, FAPs, and MVF‐FAP co‐cultures in fibrin hydrogels were formed in the individual wells of 96‐well culture plates by mixing 20 µL of the cell suspension in fibrinogen media with 50 µL of 10 U/mL thrombin (2:5 ratio), resulting in 70 µL hydrogels with final concentrations of 5.7 mg/mL fibrinogen and 7.1 U/mL thrombin. Hydrogels containing MVFs, FAPs, or MVFs + FAPs were cultured in GM for 5 days to evaluate angiogenesis. All media was supplemented with 1 mg/mL aminocaproic acid to inhibit fibrinolysis. The appropriate volume of media (150 uL) was replaced every other day, and hydrogels were maintained in a humidified incubator at 37°C and 5% CO_2_. In a subset of experiments prior to co‐culture with MVFs, FAPs were fluorescently labeled with 2.5 µM ViaFluor 488 SE (30086, Biotium) in PBS for 15 min in a humidified incubator at 37°C and 5% CO_2_ to allow for tracking of FAPs over time during co‐culture experiments. After staining, FAPs were washed 2 times with GM and 3 times with 1X PBS to hydrolyze residual dye.

### 5.8 Immunofluorescence Analysis

After 5 days of culture, fibrin hydrogels containing MVFs or MVFs and FAPs were washed 3 times with 1X PBS, fixed with 4% paraformaldehyde for 2 h, and then blocked with 10% goat serum for 2 h at room temperature on an orbital shaker. For evaluation of MVF angiogenesis, gels were stained with rhodamine‐labeled Griffonia Simplicifolia Lectin I (GS Lectin I, 1:100, RL‐1102, Vector Laboratories) overnight at 4°C and washed 3 times with 1X PBS. A subset of gels containing VF488^+^ pre‐labeled FAPs was also stained with F‐Actin (R37112, Thermo Fisher Scientific) and DAPI (R37606, Thermo Fisher Scientific) according to manufacturer protocols to visualize the actin cytoskeleton and nuclei, respectively. MVF vascular networks on day 5 were imaged (3 independent, non‐overlapping images per hydrogel) with a Leica SP8 confocal microscope using a 5X objective with a zoom factor of 1.5 (1024 × 1024 resolution, line average = 16–32) and a 100 µm Z‐stack with a 10 µm step. FAP cell tracing experiments with individual MVFs on day 1 and ROIs from MVF vascular networks on day 5 were imaged using a 20X objective with a zoom factor of 2.0 (1024 × 1024 resolution, line average = 16) and a 45 µm Z‐stack with a 2.5 µm step. Quantitative analysis of MVF angiogenesis was performed by automatically thresholding images in ImageJ (U.S. National Institutes of Health) to highlight vascular structures, then by processing images using AngioTool [44]. For each image, the input parameters “Vessel diameter and intensity” and “Remove small particles” in AngioTool were manually selected to accurately outline vessel networks and minimize the background and detection of non‐vessel structures, respectively. Results from each image were automatically exported to a Microsoft Excel spreadsheet by the AngioTool program.

### 5.9 Statistical Analysis

All statistical analyses were performed in GraphPad Prism 10.5.0, using an unpaired t‐test with Welch's correction or a one‐way analysis of variance (ANOVA) with a Tukey's post‐hoc test to determine differences between groups. Statistical significance was defined as *p* < 0.05. All results are presented as mean ± standard error of the mean (SEM), where *n* represents biological replicates (3–4 independent animals). Data points are averages of measurements of at least 3 technical replicates (individual wells/hydrogels) derived from each biological replicate. Experiments were performed on separate occasions to ensure reproducibility.

## Author Contributions

**Christian M. Mendiondo**: conceptualization, investigation, writing – original draft, methodology, validation, visualization, writing – review and editing, software, formal analysis, data curation. **Andrew R. King**: writing – review and editing, investigation, methodology. **Felipe Morales**: writing – review and editing, investigation, methodology, visualization. **David Leyton‐cifuentes**: methodology, writing – review and editing, investigation. **Theodore J. Pabst**: investigation, writing – review and editing. **Eric M. Brey**: funding acquisition, conceptualization, writing – review and editing, project administration, supervision, resources, formal analysis. **Christopher R. Rathbone**: funding acquisition, conceptualization, writing – review and editing, project administration, supervision, resources, formal analysis.

## Funding

This work was partially supported by the National Institutes of Health (5SC1DK122578, to C.R.R), the National Science Foundation (CBET–2144438, to C.R.R), and the Department of Defense Military Operational Medicine Research Program (HT9425‐23‐1‐1072, to E.M.B).

## Conflicts of Interest

The authors declare no conflicts of interest.

## Data Availability

The raw data supporting the conclusions of this article will be made available by the authors, without undue reservation.

## References

[1] H. Schmalbruch and U. Hellhammer , “The Number of Nuclei in Adult Rat Muscles With Special Reference to Satellite Cells,” The Anatomical Record 189, no. 2 (1977): 169–175, 10.1002/ar.1091890204. 911042

[2] C. Christov , F. Chrétien , R. Abou‐Khalil , et al., “Muscle Satellite Cells and Endothelial Cells: Close Neighbors and Privileged Partners,” Molecular Biology of the Cell 18, no. 4 (2007): 1397–1409, 10.1091/mbc.e06-08-0693. PMC1838982 17287398

[3] R. P. Rhoads , R. M. Johnson , C. R. Rathbone , et al., “Satellite Cell‐mediated Angiogenesis in Vitro Coincides With a Functional Hypoxia‐inducible Factor Pathway,” American Journal of Physiology‐Cell Physiology 296, no. 6 (2009): C1321–C1328, 10.1152/ajpcell.00391.2008. PMC2692418 19386789

[4] M. Verma , Y. Asakura , B. S. R. Murakonda , et al., “Muscle Satellite Cell Cross‐Talk With a Vascular Niche Maintains Quiescence via VEGF and Notch Signaling,” Cell Stem Cell 23, no. 4 (2018): 530–543.e9, 10.1016/j.stem.2018.09.007. PMC6178221 30290177

[5] A. Germani , A. Di Carlo , A. Mangoni , et al., “Vascular Endothelial Growth Factor Modulates Skeletal Myoblast Function,” The American Journal of Pathology 163, no. 4 (2003): 1417–1428, 10.1016/s0002-9440(10)63499-2. PMC1868307 14507649

[6] O. Ochoa , D. Sun , S. M. Reyes‐Reyna , et al., “Delayed angiogenesis and VEGF production in CCR2−/− mice During impaired skeletal muscle regeneration,” American Journal of Physiology‐Regulatory, Integrative and Comparative Physiology 293, no. 2 (2007): R651–R661, 10.1152/ajpregu.00069.2007. 17522124

[7] C. Latroche , M. Weiss‐Gayet , L. Muller , et al., “Coupling Between Myogenesis and Angiogenesis During Skeletal Muscle Regeneration Is Stimulated by Restorative Macrophages,” Stem Cell Reports 9, no. 6 (2017): 2018–2033, 10.1016/j.stemcr.2017.10.027. PMC5785732 29198825

[8] N. Jetten , S. Verbruggen , M. J. Gijbels , M. J. Post , M. P. De Winther , and M. M. Donners , “Anti‐Inflammatory M2, but Not Pro‐inflammatory M1 Macrophages Promote Angiogenesis in Vivo,” Angiogenesis 17, no. 1 (2014): 109–118, 10.1007/s10456-013-9381-6. 24013945

[9] P. Thummarati and M. Kino‐Oka , “Effect of Co‐culturing Fibroblasts in Human Skeletal Muscle Cell Sheet on Angiogenic Cytokine Balance and Angiogenesis,” Frontiers in Bioengineering and Biotechnology 8 (2020): 578140, 10.3389/fbioe.2020.578140. PMC7542332 33072729

[10] T. Molina , P. Fabre , and N. A. Dumont , “Fibro‐adipogenic Progenitors in Skeletal Muscle Homeostasis, Regeneration and Diseases,” Open Biology 11, no. 12 (2021): 210110, 10.1098/rsob.210110. PMC8651418 34875199

[11] A. W. B. Joe , L. Yi , A. Natarajan , et al., “Muscle Injury Activates Resident Fibro/Adipogenic Progenitors That Facilitate Myogenesis,” Nature Cell Biology 12, no. 2 (2010): 153–163, 10.1038/ncb2015. PMC4580288 20081841

[12] A. Uezumi , S. Fukada , N. Yamamoto , S. Takeda , and K. Tsuchida , “Mesenchymal Progenitors Distinct From Satellite Cells Contribute to Ectopic Fat Cell Formation in Skeletal Muscle,” Nature Cell Biology 12, no. 2 (2010): 143–152, 10.1038/ncb2014. 20081842

[13] M. P. Santini , D. Malide , G. Hoffman , et al., “Tissue‐Resident PDGFRα+ Progenitor Cells Contribute to Fibrosis versus Healing in a Context‐ and Spatiotemporally Dependent Manner,” Cell Reports 30, no. 2 (2020): 555–570.e7, 10.1016/j.celrep.2019.12.045. PMC7030884 31940496

[14] E. Groppa , P. Martini , N. Derakhshan , et al., “Spatial Compartmentalization of Signaling Imparts Source‐specific Functions on Secreted Factors,” Cell Reports 42, no. 2 (2023): 112051, 10.1016/j.celrep.2023.112051. 36729831

[15] C. Lee , M. Liu , O. Agha , H. T. Kim , X. Liu , and B. T. Feeley , “Beige Fibro‐adipogenic Progenitor Transplantation Reduces Muscle Degeneration and Improves Function in a Mouse Model of Delayed Repair of Rotator Cuff Tears,” Journal of Shoulder and Elbow Surgery 29, no. 4 (2020): 719–727, 10.1016/j.jse.2019.09.021. PMC7085983 31784382

[16] Z. Wang , C. S. Lee , J. He , et al., “FAP Beige Adipogenesis in Volumetric Muscle Loss,” Preprint (2020), 10.21203/rs.3.rs-79356/v1.

[17] H. Zhang , M. Liu , H. T. Kim , B. T. Feeley , and X. Liu , “Preconditioning improves muscle regeneration After ischemia–reperfusion injury,” Journal of Orthopaedic Research 39, no. 9 (2021): 1889–1897, 10.1002/jor.24909. PMC9257970 33232533

[18] G. Dammone , S. Karaz , L. Lukjanenko , et al., “PPARγ Controls Ectopic Adipogenesis and Cross‐Talks With Myogenesis during Skeletal Muscle Regeneration,” International Journal of Molecular Sciences 19, no. 7 (2018): 2044, 10.3390/ijms19072044. PMC6073847 30011852

[19] R. Z. Lin , R. Moreno‐Luna , B. Zhou , W. T. Pu , and J. M. Melero‐Martin , “Equal Modulation of Endothelial Cell Function by Four Distinct Tissue‐specific Mesenchymal Stem Cells,” Angiogenesis 15, no. 3 (2012): 443–455, 10.1007/s10456-012-9272-2. PMC3409933 22527199

[20] S. M. Jung , J. Sanchez‐Gurmaches , and D. A. Guertin , “Brown Adipose Tissue Development and Metabolism,” Handbook of Experimental Pharmacology, (Springer, 2019): 3–36, 10.1007/164_2018_168. PMC7330484 30203328

[21] Y. Xue , N. Petrovic , R. Cao , et al., “Hypoxia‐independent Angiogenesis in Adipose Tissues During Cold Acclimation,” Cell Metabolism 9, no. 1 (2009): 99–109, 10.1016/j.cmet.2008.11.009. 19117550

[22] M. J. During , X. Liu , W. Huang , et al., “Adipose VEGF Links the White‐to‐Brown Fat Switch with Environmental, Genetic, and Pharmacological Stimuli in Male Mice,” Endocrinology 156, no. 6 (2015): 2059–2073, 10.1210/en.2014-1905. PMC4430610 25763639

[23] K. Mahdaviani , D. Chess , Y. Wu , O. Shirihai , and T. R. Aprahamian , “Autocrine Effect of Vascular Endothelial Growth Factor‐A Is Essential for Mitochondrial Function in Brown Adipocytes,” Metabolism 65, no. 1 (2016): 26–35, 10.1016/j.metabol.2015.09.012. PMC4684900 26683794

[24] B. T. Feeley , M. Liu , C. B. Ma , et al., “Human Rotator Cuff Tears Have an Endogenous, Inducible Stem Cell Source Capable of Improving Muscle Quality and Function after Rotator Cuff Repair,” The American Journal of Sports Medicine 48, no. 11 (2020): 2660–2668, 10.1177/0363546520935855. PMC9262007 32730704

[25] C. Qi , X. Song , H. Wang , Y. Yan , and B. Liu , “The Role of Exercise‐induced Myokines in Promoting Angiogenesis,” Frontiers in Physiology 13 (2022): 981577, 10.3389/fphys.2022.981577. PMC9459110 36091401

[26] C. A. Alcazar , C. Hu , T. A. Rando , N. F. Huang , and K. H. Nakayama , “Transplantation of Insulin‐Like Growth Factor‐1 Laden Scaffolds Combined With Exercise Promotes Neuroregeneration and Angiogenesis in a Preclinical Muscle Injury Model,” Biomaterials Science 8, no. 19 (2020): 5376–5389, 10.1039/d0bm00990c. PMC7531607 32996916

[27] J. Zhu , Y. Li , A. Lu , et al., “Follistatin Improves Skeletal Muscle Healing After Injury and Disease Through an Interaction With Muscle Regeneration, Angiogenesis, and Fibrosis,” The American Journal of Pathology 179, no. 2 (2011): 915–930, 10.1016/j.ajpath.2011.04.008. PMC3157209 21689628

[28] M. W. Laschke , E. Kontaxi , C. Scheuer , A. Heß , P. Karschnia , and M. D. Menger , “Insulin‐Like growth factor 1 stimulates the angiogenic activity of adipose tissue–derived microvascular fragments,” Journal of Tissue Engineering 10 (2019): 2041731419879837, 10.1177/2041731419879837. PMC6767710 31632630

[29] M. W. Laschke and M. D. Menger , “Adipose Tissue‐derived Microvascular Fragments: Natural Vascularization Units for Regenerative Medicine,” Trends in Biotechnology 33, no. 8 (2015): 442–448, 10.1016/j.tibtech.2015.06.001. 26137863

[30] M. W. Laschke and M. D. Menger , “The Simpler, the Better: Tissue Vascularization Using the Body's Own Resources,” Trends in Biotechnology 40, no. 3 (2022): 281–290, 10.1016/j.tibtech.2021.07.002. 34404555

[31] M. W. Laschke and M. D. Menger , “Microvascular Fragments in Microcirculation Research and Regenerative Medicine,” Tissue Engineering Part B: Reviews 28, no. 5 (2022): 1109–1120, 10.1089/ten.TEB.2021.0160. 34731017

[32] Y. Gao , C. Liang , B. Yang , L. Liao , and X. Su , “Application and Mechanism of Adipose Tissue‐Derived Microvascular Fragments in Tissue Repair and Regeneration,” Biomolecules 15, no. 3 (2025): 422, 10.3390/biom15030422. PMC11939927 40149958

[33] G. T. Park , J. K. Lim , E.‐B. Choi , et al., “Transplantation of Adipose Tissue‐derived Microvascular Fragments Promotes Therapy of Critical Limb Ischemia,” Biomaterials Research 27, no. 1 (2023): 70, 10.1186/s40824-023-00395-6. PMC10350266 37455318

[34] A. Uezumi , T. Ito , D. Morikawa , et al., “Fibrosis and Adipogenesis Originate From a Common Mesenchymal Progenitor in Skeletal Muscle,” Journal of Cell Science 124 (2011): 3654–3664, 10.1242/jcs.086629. 22045730

[35] F. Trensz , S. Haroun , A. Cloutier , M. V. Richter , and G. Grenier , “A Muscle Resident Cell Population Promotes Fibrosis in Hindlimb Skeletal Muscles of mdx Mice Through the Wnt Canonical Pathway,” American Journal of Physiology‐Cell Physiology 299, no. 5 (2010): C939–C947, 10.1152/ajpcell.00253.2010. 20810909

[36] C. Latroche , B. Matot , A. Martins‐Bach , et al., “Structural and Functional Alterations of Skeletal Muscle Microvasculature in Dystrophin‐Deficient Mdx Mice,” The American Journal of Pathology 185, no. 9 (2015): 2482–2494, 10.1016/j.ajpath.2015.05.009. 26193666

[37] R. P. Rhoads , K. L. Flann , T. R. Cardinal , C. R. Rathbone , X. Liu , and R. E. Allen , “Satellite Cells Isolated From Aged or Dystrophic Muscle Exhibit a Reduced Capacity to Promote Angiogenesis in Vitro,” Biochemical and Biophysical Research Communications 440, no. 3 (2013): 399–404, 10.1016/j.bbrc.2013.09.085. 24070607

[38] J. Farup , J. Just , F. de Paoli , et al., “Human skeletal muscle CD90+ fibro‐adipogenic progenitors are associated With muscle degeneration in type 2 diabetic patients,” Cell Metabolism 33, no. 11 (2021): 2201–2214.e10, 10.1016/j.cmet.2021.10.001. PMC9165662 34678202

[39] B. B. L. Groen , H. M. Hamer , T. Snijders , et al., “Skeletal Muscle Capillary Density and Microvascular Function Are Compromised With Aging and Type 2 Diabetes,” Journal of Applied Physiology 116, no. 8 (2014): 998–1005, 10.1152/japplphysiol.00919.2013. 24577061

[40] L. J. I. Te , C. Doherty , J. Correa , and J. Batt , “Identification, Isolation, and Characterization of Fibro‐Adipogenic Progenitors (FAPs) and Myogenic Progenitors (MPs) in Skeletal Muscle in the Rat,” Journal of Visualized Experiments, no. 172 (2021): 1–32, 10.3791/61750. 34180887

[41] F. M. Acosta , K. Stojkova , E. M. Brey , and C. R. Rathbone , “A Straightforward Approach to Engineer Vascularized Adipose Tissue Using Microvascular Fragments,” Tissue Engineering Part A 26, no. 15–16 (2020): 905–914, 10.1089/ten.TEA.2019.0345. PMC7462025 32070226

[42] F. M. Acosta , K. Stojkova , J. Zhang , et al., “Engineering Functional Vascularized Beige Adipose Tissue From Microvascular Fragments of Models of Healthy and Type II Diabetes Conditions,” Journal of Tissue Engineering 13 (2022): 20417314221109337, 10.1177/20417314221109337. PMC9248044 35782994

[43] F. M. Acosta , M. A. Gonzalez Porras , K. Stojkova , S. Pacelli , C. R. Rathbone , and E. M. Brey , “Three‐Dimensional Culture of Vascularized Thermogenic Adipose Tissue From Microvascular Fragments,” Journal of Visualized Experiments 192 (2023): 64650, 10.3791/64650. PMC12124870 36806034

[44] E. Zudaire , L. Gambardella , C. Kurcz , and S. Vermeren , “A Computational Tool for Quantitative Analysis of Vascular Networks,” PLoS ONE 6, no. 11 (2011): 27385, 10.1371/journal.pone.0027385. PMC3217985 22110636

